# Metastasis Patterns and Prognosis of Octogenarians with NSCLC: A Population-based Study

**DOI:** 10.14336/AD.2019.0414

**Published:** 2020-02-01

**Authors:** Yu Gu, Junhua Zhang, Zhirui Zhou, Di Liu, Hongcheng Zhu, Junmiao Wen, Xinyan Xu, Tianxiang Chen, Min Fan

**Affiliations:** ^1^Department of Radiation Oncology, Fudan University Shanghai Cancer Center, Shanghai 200032, China.; ^2^Department of Oncology, Shanghai Medical College, Fudan University, Shanghai 200032, China.; ^3^Shanghai Lung Cancer Center, Shanghai Chest Hospital, Shanghai Jiao Tong University, Shanghai 200030, China.

**Keywords:** NSCLC, octogenarian, age-related, metastasis, prognosis, cancer, lung

## Abstract

Non-small cell lung cancer (NSCLC) is the most common cancer and the leading cause of cancer-related deaths worldwide. Age at diagnosis of advanced NSCLC is much older, but studies describing the practice patterns for octogenarians with distant metastasis NSCLC are limited. A retrospective, population-based study using national representative data from the Surveillance, Epidemiology, and End Results (SEER) program was conducted to evaluate 34 882 NSCLC patients with extrathoracic metastases from 2010 to 2013. Patients were classified into three groups (older group: ≥80 yrs, middle-aged group: 60-79 yrs, and younger group: ≤59 yrs). The role of different age at diagnosis of NSCLC in metastasis patterns was investigated, and survival of different age groups of metastatic NSCLC was assessed. The analysis revealed that older patients were more likely to only have bone or liver metastasis (p< 0.001), but less likely to have brain only metastasis (p<0.001) and multiple metastatic sites (p< 0.001) than other two groups. Age at diagnosis was an independent risk factor for different metastasis types. Older group had the worst overall survival (p<0.001) and cancer-specific survival (p<0.001). Furthermore, older age patients with only bone metastasis had the best cancer specific survival (p<0.05) while younger patients with only brain metastasis had the best prognosis (p<0.001). Over 60% octogenarians with metastatic NSCLC did not receive anti-cancer therapy and had the highest rate of cancer deaths among all patients. Our results may help clinicians make positive decisions regarding personalized treatment of metastatic NSCLC in the elderly.

Lung cancer remains a serious health issue and will account for about 30% of total cancer-related deaths in the near future [1]. Non-small cell lung cancer (NSCLC) represents more than 80% of lung cancer cases [1]. Up to 70% of NSCLC patients are diagnosed at advanced stages, of which the median overall 5-year survival rate is substantially low, at 4% to 6% [2, 3]. Distant metastases have been the main cause of mortality among lung cancer patients and the most common distant metastatic sites included bone, brain and liver [4]. Approximately, more than 25% of lung cancer patients are over 80 years old by 2016 [1]. By 2030, nearly 20% of Americans will be 65 or older, which is a four-fold increase since 1930 [5]. In current practice, older patients are always underrepresented in clinical trials and might be undertreated [6]. The physical and physiologic function decline associated with aging may be the reasons lead to lower willingness of both patients and doctors to pursue aggressive therapy [7]. A study of stage III NSCLC found that approximately 62.7% of patients aged over 80 years did not receive any cancer-directed care. These patients who received standard radiotherapy and chemotherapy had improved overall survival compared with those who did not [7]. Similarly, another study about elder patients (>=66 years) with locally advanced NSCLC who received combined modality therapy from 1997 to 2002 in SEER database also showed survival benefits associated[8]. However, to the best of our knowledge, limited population-based studies exist describing the role of age in metastatic NSCLC heterogeneity [9].

By using the Surveillance, Epidemiology, and End Results (SEER) database, we analyzed distant metastasis patterns and prognosis of different age groups especially octogenarians in a large cohort of NSCLC population. According to age, patients were divided into older group, middle-aged group and younger group. To clear the controversy about survival benefit of anti-cancer therapy for older patients, both overall survival and cancer-specific survival of three age groups with metastasis to different single organ or combination of multiple organs were compared. Many important clinicopathological parameters in addition to age were also included in the analysis, such as race, gender, histological grades, T stage, N stage, and treatment, which known to contribute individually to outcomes. Prognosis of patients of different metastasis type might be an important clue for future treatment decision. Our investigation showed previously unreported observations on outcomes in older NSCLC patients with extrathoracic metastases: octogenarians had a distinctive metastasis pattern, the least anti-cancer therapy and the worst survival.

## MATERIALS AND METHODS

### Ethics statement

The research data files were obtained from the SEER database using the reference number 12703-Nov2016. The data released by the SEER database do not require informed patient consent. Approval for this study was obtained from the Ethical Committee and Institutional Review Board of Fudan University Shanghai Cancer Centre (FDUSCC). The method was based on approved guidelines.

**Figure 1.**
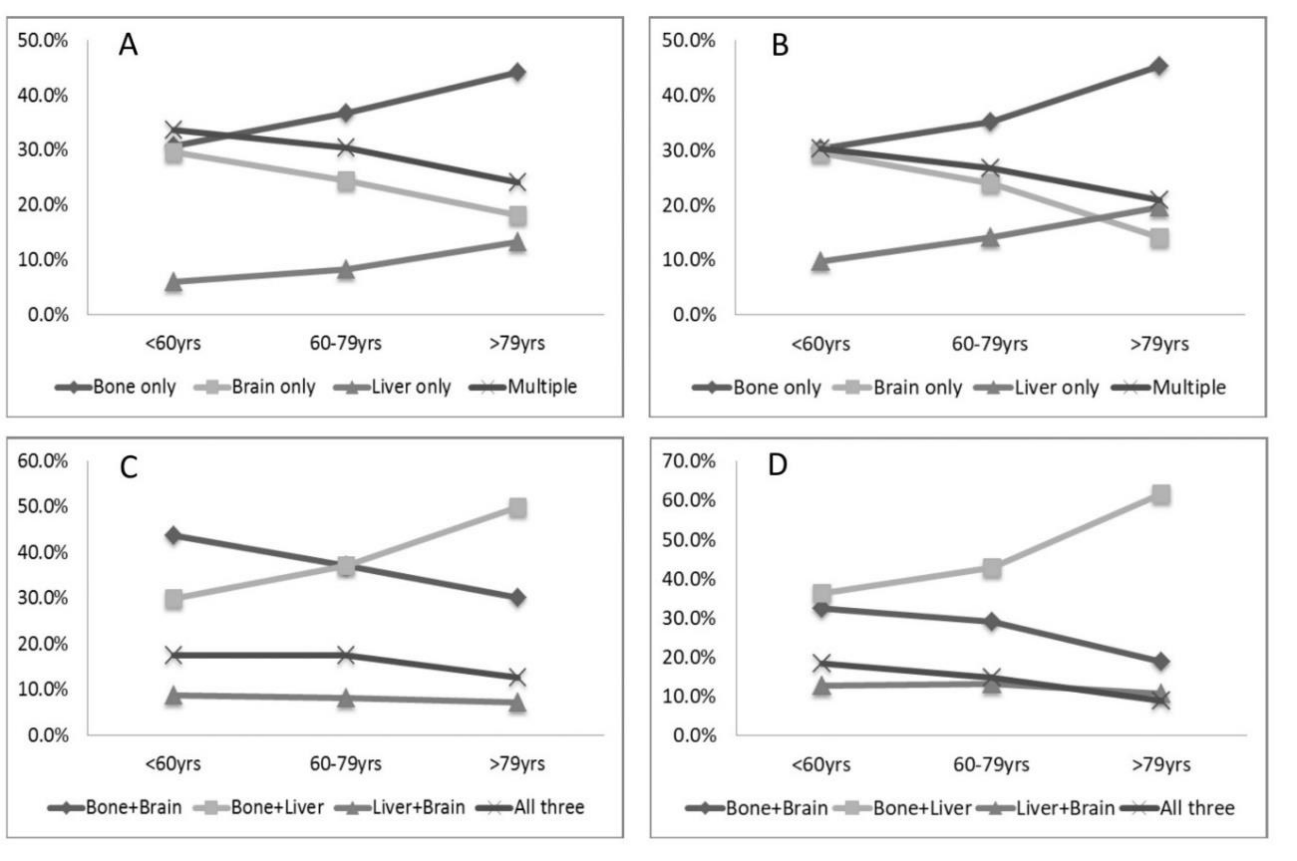
**Distant metastatic patterns of different age groups**. Metastasis patterns of adenocarcinoma (A) and non-adenocarcinoma (B) were analyzed. Different patterns of multiple metastatic sites of adenocarcinoma (C) and non- adenocarcinoma (D) were also analyzed.

**Figure 2.**
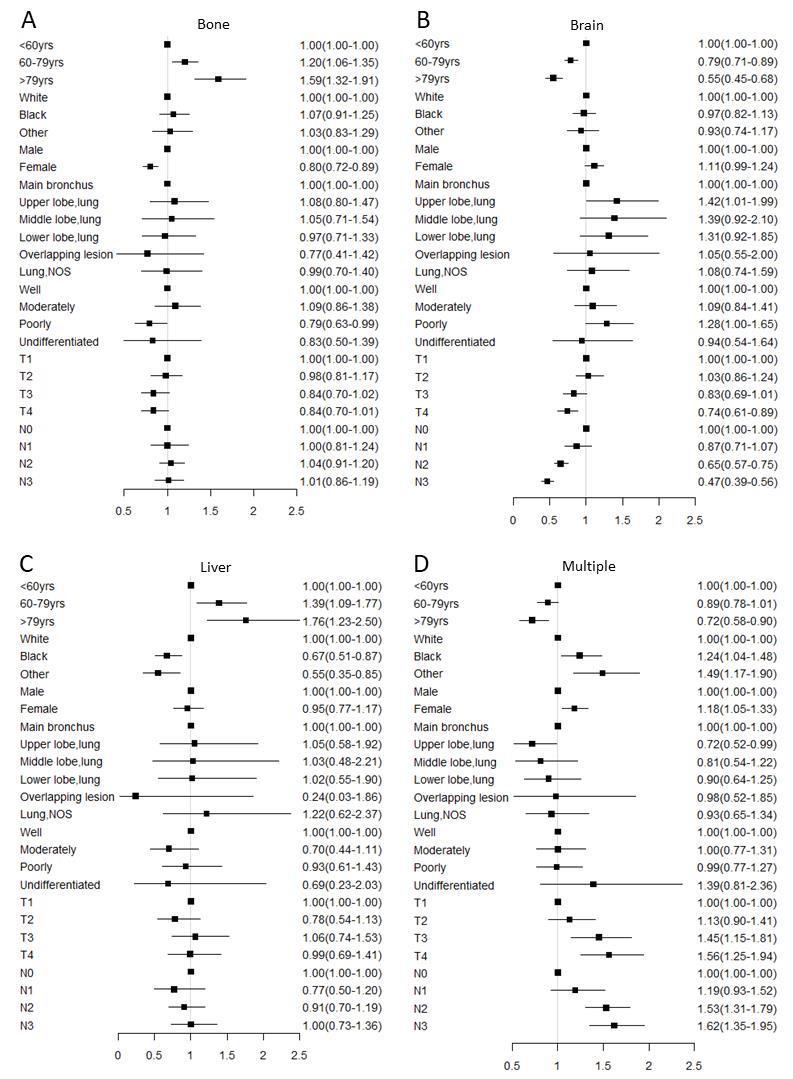
**Multivariable logistic regression analyses predicting different sites of metastasis in adenocarcinoma patients**. **(A)** only bone metastasis; (B) only brain metastasis; (C) only liver metastasis; (D) multiple metastatic sites. Abbreviation: NOS= not otherwise specified.

**Figure 3.**
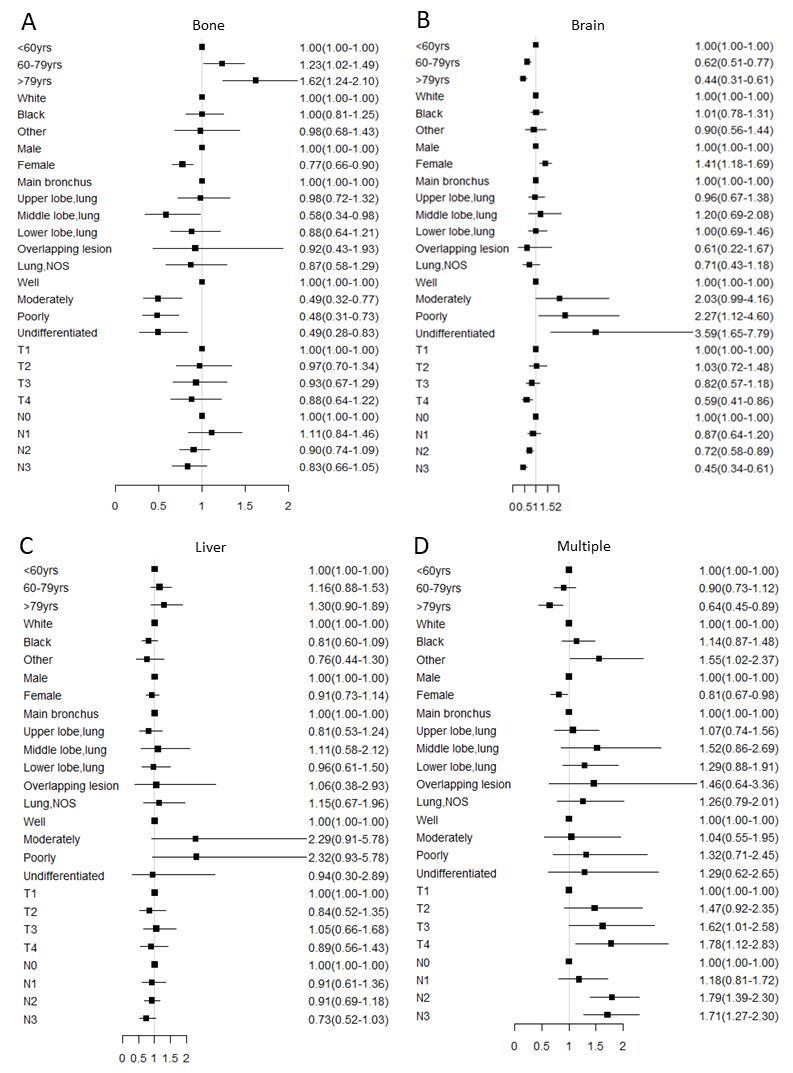
**Multivariable logistic regression analyses predicting different sites of metastasis in nonadenocarcinoma patients**. **(A)** Only bone metastasis; (B) only brain metastasis; (C) only liver metastasis; (D) multiple metastatic sites. Abbreviation: NOS= not otherwise specified.

### Data collection

We used SEER * Stat 8.3.4 version to filter information and collect a representative population of patients for this research (http://seer.cancer.gov/). SEER 18 Regs Research Data was chosen to select patients, which is maintained by the National Cancer Institute and covers nearly 28% of the population in the United States. Its follow-up ended by December 31, 2013. We limited this study population based on the following criteria: age at diagnosis ≥18 years old, primary site: lung and bronchus, primary site and morphology ICD-O-3: NSCLC (8012/ 8013/ 8014/ 8046/ 8052/ 8070-8078/ 8083/ 8084/ 8140/ 8141/ 8143/ 8144/ 8146/ 8147/ 8250-8255/ 8260/ 8310/ 8323/ 8481/ 8560), malignant behavior, microscopically confirmed primary lung cancer, only one malignant primary tumor, diagnosis between 2010 and 2013, and de novo M1b (AJCC 7th edition) patients. Finally, a total of 34 882 patients were included.

### Statistical analysis

All patients were divided into three groups: ≥80 yrs, 60-79 yrs and ≤59 yrs. Demographic and clinical characteristics were compared for patients of different age groups using one-way analysis of variance for continuous variables and Pearson’s Chi-square test statistic for categorical variables. The Kaplan-Meier analyses were used to generate the survival curves and the Log Rank test was applied to analyze the differences among the curves. Comparative risk factors of overall survival (OS) and lung cancer-specific survival (CSS) were identified using univariate and multivariate Cox regression models. OS was defined as the time from lung cancer diagnosis to death due to any cause and CSS from lung cancer diagnosis to death due to lung cancer. The association of clinicopathologic factors and the sites of distant metastases were modeled with logistic regression analysis. Both univariate and multivariate odds ratios (ORs) and 95% confidence intervals (CIs) were calculated for each model. All statistical tests were two-sided, and a *p*-value < 0.05 was defined as statistically significant. Forest plots were created using R version 3.2.3 (Vienna, Austria). All of the other calculations were performed using SPSS 21.0 statistical software (NY, USA).

## RESULTS

### Demographics

Overall, 34 882 NSCLC patients with synchronous extrathoracic metastases were included, among which 4260 (12.2%) patients were diagnosed at the age of ≥ 80 yrs, 20625 (59.1%) patients 60-79 yrs, and 9997 (28.7%) patients under 59 yrs. The median age at diagnosis was 66 yrs and the overall median follow-up time was 6.4 months. Statistically significant differences of clinicopathological characteristics among different age groups with adenocarcinoma (AD) and non- adenocarcinoma (NAD) are summarized in Table 1. Specifically, in patients with AD, compared to patients in the younger and middle-aged group, the older group had a lower rate of lymph node involvement (26.5% vs. 20.9% vs. 18.2%, respectively with N0, 15.1% vs. 20.3% vs. 25.3%, respectively with N3, p< 0.001), and lower rate of radiotherapy (37.6% vs. 48.9% vs. 58.2%, respectively, p < 0.001). In patients with NAD, the older group also showed a lower rate of lymph node involvement (28.2% vs. 20.6% vs. 15.8%, respectively with N0, 13.1% vs. 18.3% vs. 25.7%, respectively with N3, p< 0.001), and lower rate of radiotherapy (36.2% vs. 47.1% vs. 57.5%, respectively, p < 0.001).

**Figure 4.**
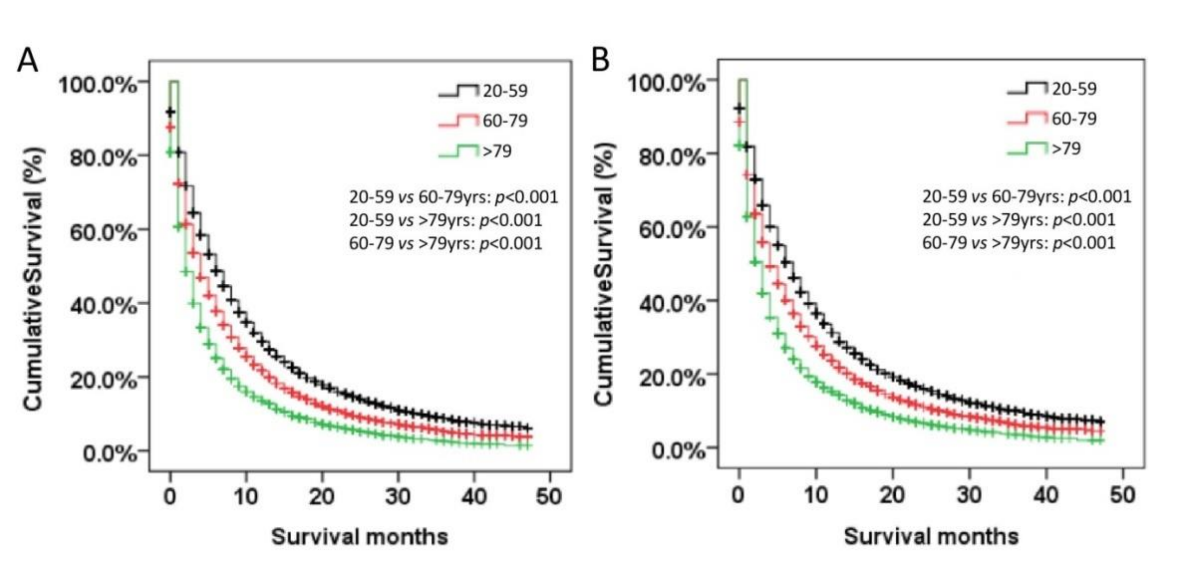
**Kaplan-Meier curve of OS (A) and CSS(B) by age groups among NSCLC patients with extrathoracic metastases**. Abbreviation: OS=overall survival, CSS=cancer-specific survival

**Table 1:** Characteristics of patients with extrathoracic metastatic AD and NAD by age groups.

	AD					NAD				
	<=59 yrs	60-79 yrs	>=80 yrs	Total	*P* value	<=59 yrs	60-79 yrs	80yrs	Total	p-value
	(n=6757, 31.1%)	(n=12500, 57.5%)	(n=2465, 11.3%)	(n = 21722)		(n =1195, 14.2%)	(n = 5296, 62.9%)	(n =1929, 22.9%)	(n = 8420)	
Race										
Black	1168(17.3%)	1471(11.8%)	154(6.2%)	2793(12.9%)	<0.001	296(15.3%)	734(13.9%)	98(08.2%)	1128(13.4%)	<0.001
White	4821(71.3%)	9771(78.2%)	2039(82.7%)	16631(76.6%)		1508(78.2%)	4243(80.1%)	1014(84.9%)	6765(80.3%)	
Other#	746(11.0%)	1227(9.8%)	268(10.9%)	2241(10.3%)		120(6.2%)	316(6.0%)	80(6.7%)	516(6.1%)	
Unknown	22(0.3%)	31(0.2%)	4(0.2%)	57(0.3%)		5(0.3%)	3(0.1%)	3(0.3%)	11(0.1%)	
Gender										
Male	3584(53.0%)	6750(54.0%)	1165(47.3%)	11499(52.9%)	<0.001	1292(67.0%)	3401(64.2%)	711(59.5%)	5404(64.2%)	<0.001
Female	3173(47.0%)	5750(46.0%)	1300(52.7%)	10223(47.1%)		637(33.0%)	1895(35.8%)	484(40.5%)	3016(35.8%)	
Histologic grade										
Well	120(1.8%)	267(2.1%)	73(3.0%)	460(2.1%)	<0.001	23(1.2%)	71(1.3%)	19(1.6%)	113(1.3%)	0.003
Moderately	749(11.1%)	1430(11.4%)	266(10.8%)	2445(11.3%)		221(11.5%)	729(13.8%)	180(15.1%)	1130(13.4%)	
Poorly	1673(24.8%)	3065(24.5%)	518(21.0%)	5256(24.2%)		594(30.8%)	1700(32.1%)	349(29.2%)	2643(31.4%)	
Undifferentiated	47(0.7%)	59(0.5%)	11(0.4%)	117(0.5%)		61(3.2%)	150(2.8%)	19(1.6%)	230(2.7%)	
Unknown	4168(61.7%)	7679(61.4%)	1597(64.8%)	13444(61.9%)		1030(53.4%)	2646(50.0%)	628(52.6%)	4304(51.1%)	
T stage										
T0	69(1.0%)	129(1.0%)	21(0.9%)	219(1.0%)	0.014	23(1.2%)	37(0.7%)	7(0.6%)	67(0.8%)	0.09
T1	779(11.5%)	1476(11.8%)	263(10.7%)	2518(11.6%)		138(7.2%)	357(6.7%)	73(6.1%)	568(6.7%)	
T2	1518(22.5%)	3006(24.0%)	598(24.3%)	5122(23.6%)		459(23.8%)	1331(25.1%)	298(24.9%)	2088(24.8%)	
T3	1524(22.6%)	2648(21.2%)	523(21.2%)	4695(21.6%)		464(24.1%)	1351(25.5%)	312(26.1%)	2127(25.3%)	
T4	1959(29.0%)	3492(27.9%)	670(27.2%)	6121(28.2%)		620(32.1%)	1620(30.6%)	342(28.6%)	2582(30.7%)	
Tx	908(13.4%)	1749(14.0%)	390(15.8%)	3047(14.0%)		225(11.7%)	600(11.3%)	163(13.6%)	988(11.7%)	
N stage										
N0	1229(18.2%)	2612(20.9%)	654(26.5%)	4495(20.7%)	<0.001	305(15.8%)	1090(20.6%)	337(28.2%)	1732(20.6%)	<0.001
N1	474(7.0%)	1004(8.0%)	169(6.9%)	1647(7.6%)		136(7.1%)	464(8.8%)	103(8.6%)	703(8.3%)	
N2	2976(44.0%)	5488(43.9%)	1026(41.6%)	9490(43.7%)		894(46.3%)	2456(46.4%)	503(42.1%)	3853(45.8%)	
N3	1712(25.3%)	2538(20.3%)	372(15.1%)	4622(21.3%)		495(25.7%)	967(18.3%)	157(13.1%)	1619(19.2%)	
Nx	366(5.4%)	858(6.9%)	244(9.9%)	1468(6.8%)		99(5.1%)	319(6.0%)	95(7.9%)	513(6.1%)	
Treatment										
Radiotherapy	3933(58.2%)	6118(48.9%)	926(37.6%)	10977(50.5%)	<0.001	1109(57.5%)	2497(47.1%)	433(36.2%)	4039(48.0%)	<0.001
Surgery and diotherapy	151(2.2%)	144(1.2%)	6(0.2%)	301(1.4%)		45(2.3%)	83(1.6%)	6(0.5%)	134(1.6%)	
No therapy	2477(36.7%)	5916(47.3%)	1483(60.2%)	9876(45.5%)		699(36.2%)	2559(48.3%)	724(60.6%)	3982(47.3%)	
Surgery	91(1.3%)	152(1.2%)	17(0.7%)	260(1.2%)		36(1.9%)	65(1.2%)	12(1.0%)	113(1.3%)	
Unknown	105(1.6%)	170(1.4%)	33(1.3%)	308(1.4%)		40(2.1%)	92(1.7%)	20(1.7%)	152(1.8%)	

# Other races included American Indians, AK Natives, Asians and Pacific Islanders. NOS=not otherwise specified

### Metastasis patterns

Patients with single and multiple organ metastatic disease were analyzed as shown in Figure 1. As the age increased, compared with the younger and middle-aged groups, the older group had the most bone only (44.3% vs. 36.8% and 30.8%, respectively in the AD population, 45.3% vs. 35.2% and 30.3%, respectively in the NAD population, p < 0.001) and liver only metastasis (13.4% vs. 8.3% and 6.0%, respectively in the AD population, 19.7% vs. 14.2% and 9.7%, respectively in the NAD population, p< 0.001). Older patients had fewer occurrences of brain only metastatic disease compared to the middle-aged and younger groups (18.2% vs. 24.5% and 29.6%, respectively in the AD population, 14.1% vs. 23.9% and 29.6%, respectively in the NAD population, p < 0.001). We also found that older patients had lower proportion of multiple metastatic sites (24.1% in AD, 20.9% in NAD) than middle-aged (30.4% in AD, 26.7% in NAD) and younger patients (33.6% in AD, 30.4% in NAD, *p*< 0.001). Among patients with multiple metastatic sites (Fig. 1C-D), the most common combination was bone plus liver in older patients (49.9% in AD, 61.6% in NAD).

**Figure 5.**
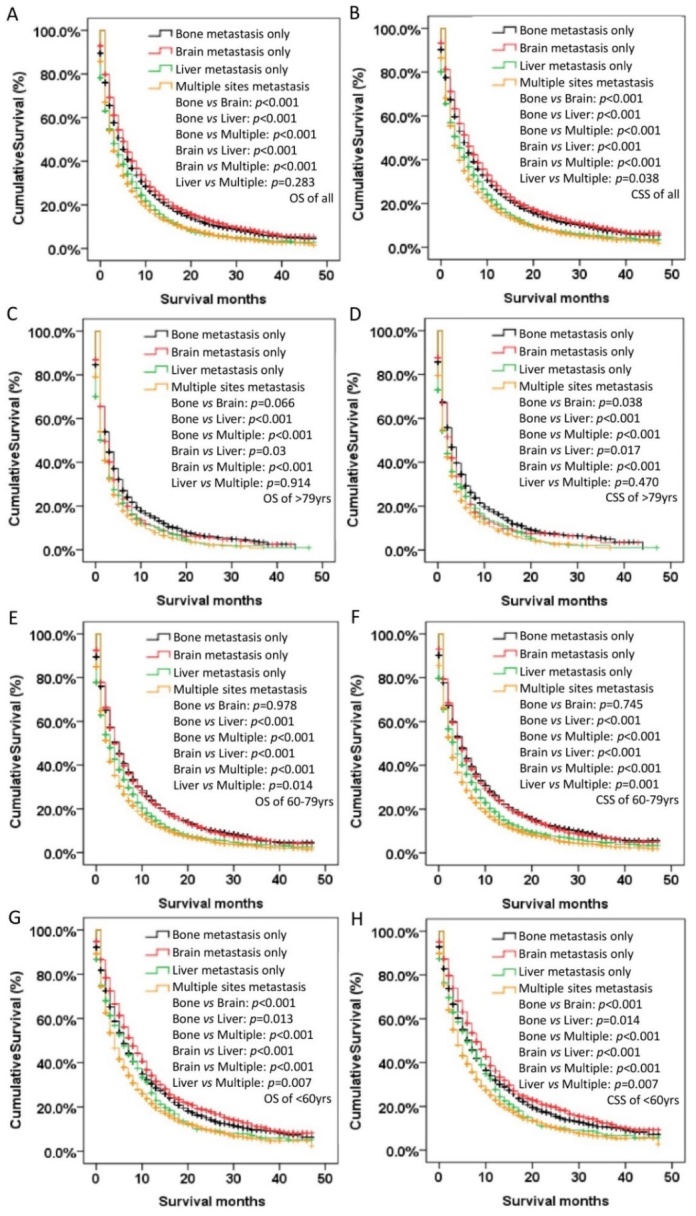
**Kaplan-Meier curve of OS and CSS according to metastasis sites in all (A-B), older (>79 yrs) (C-D), middle-aged (60-79 yrs) (E-F), and younger (<60 yrs) (G-H) patients with NSCLC**. Abbreviation: OS=overall survival, CSS=cancer-specific survival.

### Risks examined for association with different metastasis sites

In logistic regression models adjusted for race, gender, histologic grade, T stage and N stage, the odds for bone only (OR: 1.59, 95%CI: 1.32-1.91 for older patients in the AD group, OR: 1.62, 95%CI: 1.24-2.10 for older patients in the NAD group) and liver only (OR: 1.76, 95%CI: 1.23-2.50 for older patients in the AD group) metastases significantly increased with the older groups compared with the younger group (Fig. 2A,C&3A). On the other hand, relative to younger patients, there was the lowest odd ratio in older people of brain only (OR: 0.55, 95%CI: 0.45-0.68 for older patients in the AD group, OR: 0.44, 95%CI: 0.31-0.61 for older patients in the NAD group) or multiple sites metastases both in the AD and NAD population (OR: 0.72, 95%CI: 0.58-0.90 for older patients in the AD group, OR: 0.64, 95%CI: 0.45-0.89 for older patients in the NAD group) (Fig. 2B,D and 3B,D).

### Survival outcomes among age groups

Overall and lung cancer-specific survival were both compared according to the age groups (Fig. 4). Older patients had the worst OS and CSS. The mean overall survival time was 5.9, 8.6 and 11.3 months respectively in the older, middle-aged and younger group. Age at diagnosis was an independent prognostic factor for survival. Compared to younger patients, the older group had the worst OS (HR: 1.76, 95% CI: 1.63-1.92, *p*< 0.001) and CSS (HR: 1.76, 95% CI: 1.62-1.91, *p*< 0.001). Other factors associated with survival in the multivariate analysis included race, gender, location, histologic grade, N stage, treatment, and metastatic sites (*p* < 0.05) (Supplementary Table 1).

**Figure 6.**
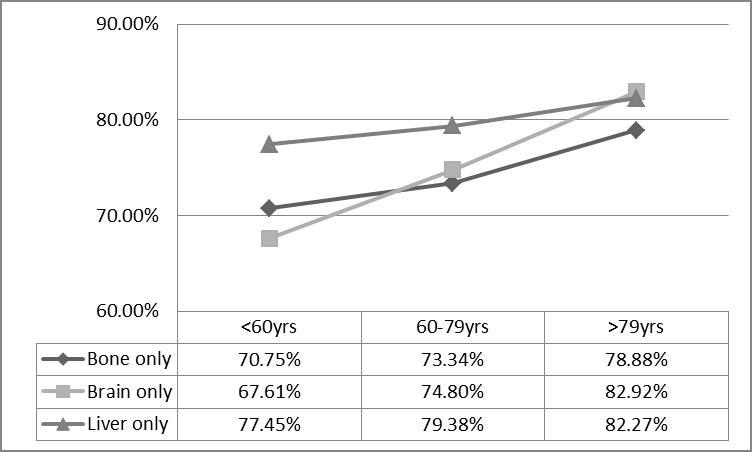
Rates of cancer death for different metastasis sites by age group.

As shown in Figure 5 and Supplementary Table2, liver only metastasis showed the worst OS and CSS in the single organ metastases population (MST:7.1 months in OS and 7.8 months in CSS, *p* < 0.001). And patients with brain only metastasis had superior prognosis compared to other sites of metastases (MST: 10.3 months in OS and 11.0 months in CSS, *p <* 0.001) (Fig. 5). Interestingly, for the older group, bone only metastasis showed significantly better CSS than other metastases including brain only metastasis (MST: 7.2 months vs. 6.1 months for the bone only subgroup and brain only subgroup, respectively,* p* < 0.05) (Fig. 5). Similar prognosis feature was seen in AD or NAD population (Supplementary Table2). The cancer death rates of NSCLC patients ≥80 yrs were higher than the other two groups, regardless of whether it was bone only, brain only or liver only metastasis (Fig. 6).

## Discussion

With extending of human life span and with the development of diagnostic technology, age at diagnosis of advanced NSCLC is older than before. Older patients with lung cancer have a distinct clinical presentation and prognosis [10, 11]. In our study, several clinicopathological parameters showed significant differences among three age groups including race, gender, histological grades, T stage, N stage, and treatment. We found that patients older than 79 years old had distinct metastasis patterns. Our data has also shown that after adjusting for race, gender, location, histologic grade, T stage and N stage, age at diagnosis was an independent risk factor with respect to NSCLC extrathoracic metastases.

As we know, the time period from the primary tumor presence to subsequent metastases is usually very long. There are complex ways for disseminated cells to interact with host organ microenvironment. Different ways may result in distinctive patterns of metastatic events. The ‘seed and soil’ hypothesis can be proposed to explain this phenomenon [12]. It was still unclear whether the different effects of age on NSCLC death and metastasis are due to the biology of primary tumor or variations of host organ response over time. Metastatic progression of lung cancer is hypothesized to be mediated by cancer factors in “seed”, including different classes of metastatic driving genes. The increased rate of gene mutation suggests that lung cancer may have distinctive metastatic patterns based on mutational profiling. EGFR mutation have been found to be associated with more frequent bone and brain metastases, whereas ALK positive was tended to have more brain metastasis than negative ones[13-16]. Genetic profiles of young and old patients were also studied. TP53, EGFR, KRAS alterations may be less likely to be found in early onset of lung cancer in older patients [17]. Turning to the “soil”, along with senescence, the internal environment function of each system is low and disordered. For example, aging is related to structural and functional alterations of human immune system, which is described as ‘immuno-senescence’. Immunosenescence is a progressive decline of immune functions and a dynamic process of adaptation[18]. Furthermore, a tumor microenvironment with enhanced fibroblast-mediated angiogenesis, stromal remodeling and inflammation is more likely to be seen in older animals [19-21]. However, the effects of the aged microenvironment on tumor progression have been largely unexplored.

Regarding impact of ethnicity in this study, we found that African-American patients with AD had a lower risk of liver metastasis but a higher risk of multiple metastases (Fig. 2). Several previous studies did demonstrate that patients of different ethnicities may show diverse metastatic patterns [22, 23]. The difference might be due to genetic diversity or social-economic factors. Insured patients might receive more early intervention of NSCLC [7, 24]. The relationship between ethnicity and metastasis requires further investigations.

According to the previous literature, younger NSCLC patients often suffer from a delayed diagnosis since doctors often consider it a benign disease at first. The time from the onset of symptoms to diagnosis was often up to four months for lung cancer patients younger than 40 years [10, 25]. These might explain why older NSCLC patients with distant metastases had fewer metastases sites and less advanced N stage than younger patients. In our Kaplan Meier analysis, older age significantly contributed to worse OS and CSS. Multiple factors can help to explain this. The poorest prognosis in older group (≥80yrs) was related to decreased physiologic reserve, reduced benefit of cancer treatment and increased risks of toxicities and death. It was reported that NSCLC patients aged over 80 years old were less likely to receive chemotherapy as initial treatment than those aged 70-79 (12.3% vs. 40.9%)[26]. Older patients were also less likely to receive local treatment such as radiation therapy or surgery in our population, which is consistent with other studies [27]. Even with distant metastases, patients can still get survival benefit from local treatment. In addition, another explanation might be that targetable genomic alterations were significantly higher among patients younger than 50 years and potential target therapy was associated with improved survival [28]. Recently, aging and disrupted rest-activity rhythm together were also found to negatively influence the survival of lung cancer patients and significantly increased their death risk, which opened up a new train of thought for us [29].

Our study also demonstrated that different metastasis patterns might lead to different survival outcomes. To be specific, the multiple site metastases group had the poorest outcomes. This may be because effective therapy is still limited for multiple site metastases. Interestingly, the bone only metastasis subgroup had the biggest population but also the longest CSS in patients over 79 years old, and the brain only metastasis group had the biggest population but also the longest OS in patients under 60 years old. These findings remind us to be more aggressive for those patients with brain only metastasis under 60 years and with bone only metastasis over 79 years. Especially for people over 79, different types of local therapies have been used for the treatment of limited metastases and proved to be effective, which including stereotactic body radiotherapy (SBRT), radiofrequency ablation and surgery[30, 31].

There were some limitations of our research. No information was provided in the SEER database regarding systemic treatment such as chemotherapy and targeted therapy, which might influence the survival. Clinical parameters such co-morbidities from the SEER database were limited, which might create some bias in our results. Limitations in the SEER database are common to most large epidemiological databanks. Despite these, the database provided valuable data for analyzing patterns in NSCLC cases with extrathoracic metastases across the United States. Additional work is needed to explore age-related changes in tumor biology and microenvironment. External factors also need to be investigated, such as poorer care and fewer aggressive treatments in the elderly. They will help us to diagnose and treat metastatic NSCLC patients in a more individualized manner.

In conclusion, our findings summarize the metastasis patterns and survival outcomes of NSCLC patients in three age groups from a large sample of the population. Over 60% patients more than 80yrs old did not receive anti-cancer therapy and these octogenarians had the highest rate of cancer deaths among all patients. Specifically, local treatment might be underrepresented in octogenarians with single organ metastasis such as bone metastasis. Our findings hopefully provide a better understanding of cancer treatment in these elderly NSCLC patients.

## Supplementary Materials

The Supplemenantry data can be found online at: www.aginganddisease.org/EN/10.14336/AD.2019.0414.
