# Supplementary material for: Metastasis Patterns and Prognosis of Octogenarians with NSCLC: A Population-based Study
Source: Aging Dis. 2020 Feb 1;11(1):82–92. doi: 10.14336/AD.2019.0414 (PMC6961775; doi:10.14336/AD.2019.0414)
Supplement: Supplementary file 1 — The Supplemenantry data can be found online at: www.aginganddisease.org/EN/10.14336/AD.2019.0414. [file AD-11-1-82-s.pdf]

SUPPLEMENTARY DATA

Metastasis Patterns and Prognosis of Octogenarians with NSCLC: A Population-based Study

Yu Gu[1,2], Junhua Zhang[1,2], Zhirui Zhou[1,2], Di Liu1,2, Hongcheng Zhu[1,2], Junmiao Wen[1,2], Xinyan Xu[1,2], Tianxiang Chen[3,*], Min Fan[1,2,*]

[1]Department of Radiation Oncology, Fudan University Shanghai Cancer Center, Shanghai 200032, China.
[2]Department of Oncology, Shanghai Medical College, Fudan University, Shanghai 200032, China.
[3]Shanghai Lung Cancer Center, Shanghai Chest Hospital, Shanghai Jiao Tong University, Shanghai 200030, China.

© 2019. Gu Y et al. Published online at http://www.aginganddisease.org/EN/10.14336/AD.2019.0414

SUPPLEMENTARY DATA

Supplementary Table 1. Univariate and multivariate analysis of OS and CSS of the study population.

	OS						CSS					
	Univariate analysis			Multivariate analysis			Univariate analysis			Multivariate analysis		
	HR	95% CI	P value	HR	95% CI	P value	HR	95% CI	P value	HR	95% CI	P value
Age												
20-59	-	-	-	-	-	-	-	-	-	-	-	-
60-79	1.28	1.24-1.31	0.000	1.28	1.21-1.35	0.000	1.26	1.22-1.30	0.000	1.28	1.21-1.35	0.000
>=80	1.73	1.66-1.80	0.000	1.76	1.63-1.92	0.000	1.71	1.65-1.78	0.000	1.76	1.62-1.91	0.000
Race												
Black	-	-	-	-	-	-	-	-	-	-	-	-
White	0.99	0.95-1.02	0.490	0.97	0.91-1.04	0.429	1.01	0.97-1.04	0.803	0.99	0.92-1.06	0.745
Other	0.70	0.66-0.73	0.000	0.66	0.59-0.73	0.000	0.70	0.67-0.74	0.000	0.67	0.60-0.75	0.000
Gender												
Male	-	-	-	-	-	-	-	-	-	-	-	-
Female	0.82	0.8-0.84	0.000	0.82	0.78-0.86	0.000	0.83	0.81-0.85	0.000	0.82	0.78-0.86	0.000
Year of diagnosis												
2010	-	-	-	-	-	-	-	-	-	-	-	-
2011	0.96	0.93-0.99	0.008	0.98	0.93-1.04	0.582	0.96	0.93-0.99	0.005	0.97	0.91-1.03	0.380
2012	0.96	0.93-0.99	0.009	0.95	0.9-1.01	0.132	0.95	0.92-0.99	0.005	0.95	0.89-1.01	0.096
2013	0.87	0.84-0.9	0.000	0.92	0.86-0.99	0.027	0.86	0.83-0.90	0.000	0.92	0.85-0.99	0.022
Location												
Main bronchus	-	-	-	-	-	-	-	-	-	-	-	-
Upper lobe,lung	0.80	0.76-0.85	0.000	0.88	0.79-0.98	0.024	0.80	0.75-0.85	0.000	0.89	0.79-0.99	0.037
Middle lobe,lung	0.79	0.73-0.85	0.000	0.88	0.75-1.02	0.096	0.79	0.73-0.86	0.000	0.88	0.75-1.03	0.113
Lower lobe,lung	0.84	0.79-0.90	0.000	0.92	0.82-1.03	0.157	0.84	0.79-0.90	0.000	0.93	0.83-1.05	0.231
Overlapping lesion	0.98	0.86-1.11	0.715	0.97	0.76-1.24	0.829	0.97	0.85-1.11	0.626	0.98	0.76-1.26	0.868
Lung,NOS	1.00	0.94-1.06	0.912	1.04	0.92-1.19	0.523	0.99	0.93-1.05	0.650	1.05	0.92-1.20	0.480
Histology												
Adenocarcinoma	-	-	-	-	-	-	-	-	-	-	-	-
Squamous cell	1.36	1.32-1.40	0.000	1.28	1.21-1.36	0.000	1.36	1.32-1.40	0.000	1.29	1.21-1.36	0.000
Adenosquamous	1.11	1.01-1.23	0.029	1.09	0.91-1.30	0.341	1.10	0.99-1.21	0.067	1.09	0.91-1.31	0.354
Large cell carcinoma	1.34	1.25-1.43	0.000	1.19	1.03-1.37	0.017	1.35	1.26-1.45	0.000	1.22	1.05-1.40	0.008
BAC	0.75	0.64-0.89	0.001	0.92	0.65-1.29	0.626	0.72	0.60-0.85	0.000	0.89	0.63-1.27	0.533
NOS，NSCLC	1.32	1.28-1.37	0.000	1.25	1.16-1.34	0.000	1.34	1.29-1.38	0.000	1.25	1.16-1.34	0.000
Histologic grade												
Well	-	-	-	-	-	-	-	-	-	-	-	-
Moderately	1.21	1.09-1.35	0.000	1.09	0.96-1.24	0.200	1.20	1.08-1.34	0.001	1.08	0.95-1.24	0.232
Poorly	1.59	1.44-1.76	0.000	1.35	1.19-1.53	0.000	1.59	1.43-1.76	0.000	1.36	1.19-1.54	0.000
Undifferentiated	1.88	1.63-2.16	0.000	1.56	1.30-1.87	0.000	1.91	1.66-2.21	0.000	1.58	1.30-1.90	0.000
T stage												
T0	-	-	-	-	-	-	-	-	-	-	-	-
T1	0.94	0.82-1.07	0.364	0.86	0.52-1.42	0.551	0.92	0.80-1.05	0.220	0.82	0.50-1.36	0.451
T2	1.18	1.03-1.34	0.013	1.06	0.64-1.75	0.817	1.16	1.02-1.33	0.024	1.01	0.61-1.66	0.980
T3	1.33	1.17-1.51	0.000	1.18	0.72-1.94	0.517	1.31	1.15-1.49	0.000	1.12	0.68-1.85	0.651
T4	1.36	1.20-1.55	0.000	1.24	0.75-2.03	0.401	1.35	1.19-1.54	0.000	1.19	0.72-1.96	0.492
N stage												
N0	-	-	-	-	-	-	-	-	-	-	-	-
N1	1.10	1.04-1.15	0.000	1.03	0.94-1.13	0.474	1.11	1.05-1.17	0.000	1.04	0.95-1.14	0.423
N2	1.20	1.16-1.24	0.000	1.13	1.06-1.20	0.000	1.21	1.17-1.25	0.000	1.14	1.07-1.21	0.000
N3	1.15	1.11-1.19	0.000	1.14	1.06-1.23	0.000	1.16	1.12-1.21	0.000	1.17	1.08-1.26	0.000
Treatment												
Radiotherapy	-	-	-	-	-	-	-	-	-	-	-	-
Surgery and	0.51	0.46-0.58	0.000	0.56	0.48-0.65	0.000	0.50	0.44-0.56	0.000	0.55	0.47-0.65	0.000
No therapy	1.27	1.24-1.30	0.000	1.27	1.21-1.34	0.000	1.25	1.22-1.28	0.000	1.25	1.19-1.32	0.000

© 2019. Gu Y et al. Published online at http://www.aginganddisease.org/EN/10.14336/AD.2019.0414

Surgery	0.60	0.53-0.68	0.000	0.73	0.60-0.88	0.001	0.56	0.49-0.64	0.000	0.66	0.54-0.81	0.000
Sites of metastasis												
Only bone	-	-	-	-	-	-	-	-	-	-	-	-
Only brain	0.92	0.89-0.96	0.000	1.16	1.09-1.23	0.000	0.93	0.90-0.96	0.000	1.15	1.08-1.23	0.000
Only liver	1.27	1.21-1.33	0.000	1.02	0.93-1.10	0.716	1.26	1.21-1.33	0.000	1.03	0.94-1.12	0.572
Multiple	1.31	1.27-1.36	0.000	1.42	1.34-1.51	0.000	1.34	1.29-1.38	0.000	1.43	1.35-1.52	0.000

Abbreviation: HR=hazard ratios, CI=confidence interval, NOS= not otherwise specified, BAC=bronchioloalveolar carcinoma, NSCLC=non-small cell lung cancer, OS=overall survival, CSS= cancer-specific survival.

Supplementary Table 2. The primers for Quantitative Real-Time PCR were listed as followed.

AD		Bone only	Brain only	Liver only	Multiple	Total
OS of <60yrs	MST	12.8	14.6	11.3	9.6	12.5
OS of 60-79 yrs	MST	10.7	10.5	8.2	7.0	9.7
OS of >79 yrs	MST	7.4	6.5	5.2	4.8	6.7
CSS of <60 yrs	MST	13.3	15.2	11.7	10.1	13.1
CSS of 60-79 yrs	MST	11.5	11.3	9.2	7.5	10.5
CSS of >79 yrs	MST	8.2	7.2	5.8	5.1	7.4
Total OS	MST	10.9	11.7	8.4	7.7	
Total CSS	MST	11.6	12.4	9.2	8.2	
NAD		Bone only	Brain only	Liver only	Multiple	Total
OS of <60yrs	MST	8.6	9.9	7.5	5.6	8.7
OS of 60-79 yrs	MST	6.6	7.1	5.7	4.4	6.9
OS of >79 yrs	MST	5.7	4.3	3.7	3.0	5.2
CSS of <60 yrs	MST	9.0	10.4	7.9	5.9	9.2
CSS of 60-79 yrs	MST	7.3	7.6	6.2	4.7	7.5
CSS of >79 yrs	MST	6.2	4.6	4.1	3.1	5.7
Total OS	MST	6.9	7.3	5.7	4.6	
Total CSS	MST	7.5	8.2	6.2	4.9	
ALL		Bone only	Brain only	Liver only	Multiple	Total
OS of <60yrs	MST	11.6	13.1	9.9	8.7	
OS of 60-79 yrs	MST	9.3	9.2	6.9	6.1	
OS of >79 yrs	MST	6.5	5.6	4.5	4.2	
CSS of <60 yrs	MST	12.2	13.8	10.4	9.2	
CSS of 60-79 yrs	MST	10.1	9.9	7.7	6.6	
CSS of >79 yrs	MST	7.2	6.1	5.1	4.4	
Total OS	MST	9.5	10.3	7.1	4.8	
Total CSS	MST	10.2	11.0	7.8	7.2	

Abbreviation: MST= median survival time, OS=overall survival, CSS= cancer-specific survival.

© 2019. Gu Y et al. Published online at http://www.aginganddisease.org/EN/10.14336/AD.2019.0414
